# Effect of Breath Holding on Spleen Volume Measured by Magnetic Resonance Imaging

**DOI:** 10.1371/journal.pone.0068670

**Published:** 2013-06-26

**Authors:** Yusuke Inoue, Ai Nakajima, Shinya Mizukami, Hirofumi Hata

**Affiliations:** 1 Department of Diagnostic Radiology, Kitasato University School of Medicine, Sagamihara, Kanagawa, Japan; 2 Department of Radiology, Kitasato University Hospital, Sagamihara, Kanagawa, Japan; National Institute of Radiological Sciences, Japan

## Abstract

**Objective:**

Ultrasonographic studies have demonstrated transient reduction in spleen volume in relation to apnea diving. We measured spleen volume under various respiratory conditions by MR imaging to accurately determine the influence of ordinary breath holding on spleen volumetry.

**Materials and Methods:**

Twelve healthy adult volunteers were examined. Contiguous MR images of the spleen were acquired during free breathing and during respiratory manipulations, including breath holding at the end of normal expiration, breath holding at deep inspiration, and the valsalva maneuver, and spleen volume was measured from each image set based on the sum-of-areas method. Acquisition during free breathing was performed with respiratory triggering. The duration of each respiratory manipulation was 30 s, and five sets of MR images were acquired serially during each manipulation.

**Results:**

Baseline spleen volume before respiratory manipulation was 173.0 ± 79.7 mL, and the coefficient of variance for two baseline measures was 1.4% ± 1.6%, suggesting excellent repeatability. Spleen volume decreased significantly just after the commencement of respiratory manipulation, remained constant during the manipulation, and returned to the control value 2 min after the cessation of the manipulation, irrespective of manipulation type. The percentages of volume reduction were 10.2% ± 2.9%, 10.2% ± 3.5%, and 13.3% ± 5.7% during expiration breath holding, deep-inspiration breath holding, and the valsalva maneuver, respectively, and these values did not differ significantly.

**Conclusions:**

Spleen volume is reduced during short breath-hold apnea in healthy adults. Physiological responses of the spleen to respiratory manipulations should be considered in the measurement and interpretation of spleen volume.

## Introduction

 Splenomegaly occurs in various disorders, such as hematological diseases, malignant neoplasms, liver diseases, infection, and autoimmune disorders. The quantitative measurement of spleen size using an imaging method plays an important role in detection, severity assessment, and monitoring of splenomegaly. Spleen size can be evaluated by ultrasonography [1–4], computed tomography (CT) [5–8], magnetic resonance (MR) imaging [9–13], or single-photon emission CT [14,15]. Among these modalities, ultrasonographic measurement of spleen length is the most popular because of its wide availability and convenience, and spleen volume may be calculated from three dimensions using a predefined formula [4]. However, ultrasonographic assessment has technical limitations that impair accuracy and reproducibility. In general, ultrasonography is highly operator dependent. Visualization of the spleen requires sufficient caudal movement at deep inspiration to avoid overlapping of the lung and ribs, and may be hampered by a suboptimal acoustic window. Spleen length is not necessarily a good representation of volume because of large variability in spleen shape. CT and MR imaging permit the estimation of organ volume from thin, contiguous slices covering the organ using the sum-of-areas technique [5,16]. These methods are less operator dependent than ultrasonography and provide accurate, reproducible measures of organ volume.

 The spleen serves as a reservoir of blood cells and releases them into the central circulation in response to physiological stresses such as exercise and apnea [17], resulting in increased circulating hemoglobin and decreased spleen volume. Breath-hold apnea has been shown to reduce spleen volume [18–22]. Imaging assessment of spleen volume may be performed under various respiratory conditions. In CT measurement of spleen volume, images are obtained during a single breath hold at deep inspiration. MR images of the spleen may be acquired during free breathing or during a single breath hold at the end of normal expiration or at deep inspiration. The duration of breath holding also varies among imaging methods and facilities. Considering the influence of breath holding on spleen volume, differences in respiratory conditions may introduce variability into estimated spleen volume.

 Previous studies [18–22] have used ultrasonography to assess alterations in spleen volume caused by breath-hold apnea, which may limit the reliability of the obtained results. It should be especially difficult to accurately measure spleen volume during free breathing for baseline assessment. In addition, the previous studies primarily aimed to assess physiology associated with diving, and experimental conditions were designed to simulate diving: enrolment of divers [18,19,21,22], long breath holding [18–21], and immersion of the whole body or face in water [18,19,21]. Spleen volumes were measured before and after breath holding but not during breath holding in most studies [18–21]. In the present study, we measured spleen volume before, during, and after short breath holding in healthy volunteers using MR imaging. In contrast to CT, MR imaging permits repeated measurement because of the absence of radiation exposure. Spleen volume during free breathing can be measured in combination with a respiratory triggering technique. The aim of this study was to accurately determine the influence of ordinary breath holding on spleen volume in non-divers.

## Materials and Methods

### Subjects

 The study subjects were 12 healthy adult volunteers (6 men and 6 women), aged 31.0 ± 5.1 (mean ± SD) years. They had no history of chronic diseases or smoking and no contraindications to MR imaging. No experienced diver was included. Before participation, the ability of 30-s breath holding at expiration was confirmed. The study protocol was approved by the institutional review board of Kitasato University School of Medicine and Hospital (approval number B12-03), and written informed consent was obtained from all subjects before participation.

### Imaging Procedures

MR images of the spleen were acquired during free breathing, breath holding at the end of normal expiration, breath holding at deep inspiration, and the Valsalva maneuver, and spleen volume was measured from each image set. A 1.5-T clinical scanner (Signa HDxt; GE Healthcare, Milwaukee, WI) and a 12-channel phased-array coil were used. Thirteen contiguous slices of 8-mm thickness were obtained in the sagittal or coronal plane to cover the entire spleen using a two-dimensional fast imaging employing steady-state acquisition (FIESTA) sequence with fat saturation. Although the axial images are the most popular in abdominal MR imaging, the superior-inferior dimension of the spleen is relatively long. We used sagittal or coronal images to reduce the number of slices and slice thickness. Scan parameters were as follows: repetition time, 3.0 ms; echo time, 1.2 ms; flip angle, 45°; number of excitations, 1; bandwidth, ±125 kHz; field-of-view (FOV), 400 mm; acquisition matrix, 200 × 200; phase FOV, 0.9; and reconstruction matrix, 512 × 512. A parallel imaging technique, the array spatial sensitivity encoding technique (ASSET), was used with a reduction factor of 2. Acquisition during free breathing was performed with respiratory triggering, and one slice was acquired for a 266-ms data acquisition window at the end of expiration in one respiratory cycle. Without respiratory triggering, acquisition time for the entire image set comprising 13 slices was 6 s.

 On the day before MR imaging, the subjects practiced respiratory manipulations after detailed explanation. MR imaging was performed in the evening after a 4-h or longer fast. A bellows was placed around the abdomen to monitor respiratory motion, and a pulse oximeter (4500 MRI pulse oximeter; In vivo, Orlando, FL) was attached to the fingertip to monitor peripheral oxygen saturation and pulse rate. First, the spleen was imaged in the sagittal and coronal planes during free breathing. The better plane for demarcation of the spleen was selected visually from the sagittal and coronal planes, and subsequent acquisitions were performed in that plane.

**Figure 1:**
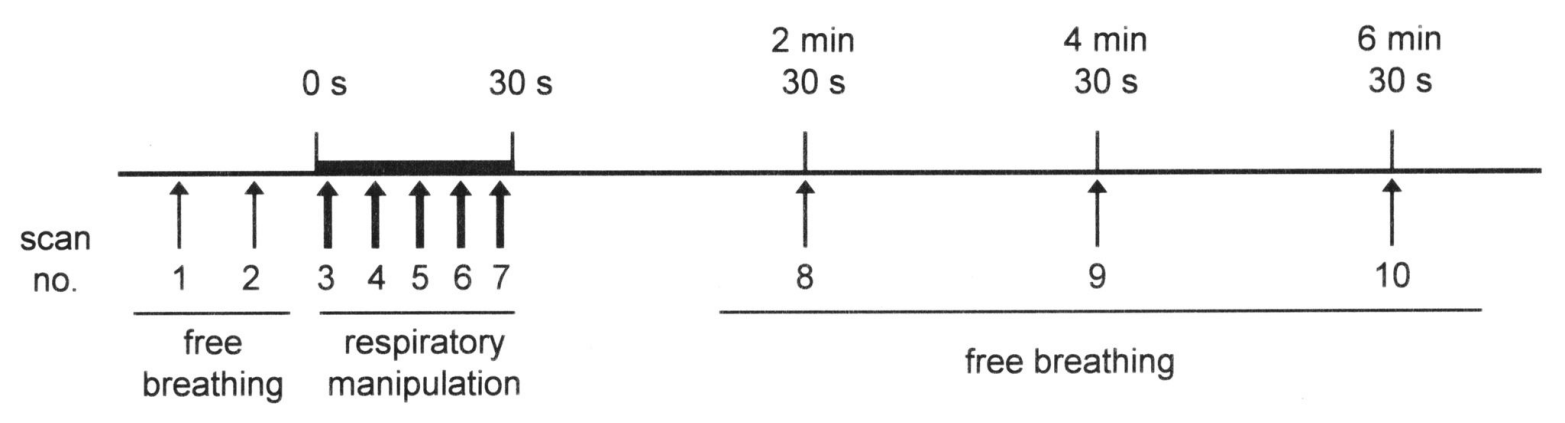
Diagram of imaging protocol. Thin and thick arrows indicate MR imaging with and without respiratory triggering, respectively. The presented course was repeated three times serially with different respiratory manipulations in one examination. The respiratory manipulation series used was breath holding at expiration, followed by breath holding at deep inspiration and the Valsalva maneuver in six subjects; and breath holding at deep inspiration, followed by breath holding at expiration and the Valsalva maneuver in the other six subjects.

Figure 1 presents a diagram showing the time course of respiratory manipulation and imaging. First, imaging during free breathing was performed twice in the selected plane. The subjects were then instructed to hold their breath for 30 s at the end of normal expiration; starting immediately after the commencement of the breath hold, five sets of MR images were acquired serially during the 30-s period. Images during free breathing were acquired at 2, 4, and 6 min after the release of breath holding. Oxygen saturation and pulse rate were recorded at each imaging. Following image acquisition at 6 min after breath-hold release, oxygen saturation and pulse rate were confirmed to be comparable with those before respiratory manipulation. Thereafter, serial imaging during deep-inspiration breath holding was performed for 30 s, and imaging during free breathing was performed 2, 4, and 6 min after the release of breath holding. Finally, serial imaging during the Valsalva maneuver was performed for 30 s, and imaging during free breathing was performed 2, 4, and 6 min after the cessation of the Valsalva maneuver. Imaging during deep-inspiration breath holding preceded imaging during expiration breath holding in three men and three women. One examination included three respiratory manipulations, and two image sets acquired during free breathing just before each respiratory manipulation were regarded as control image sets for the manipulation.

### Data Analysis

**Figure 2:**
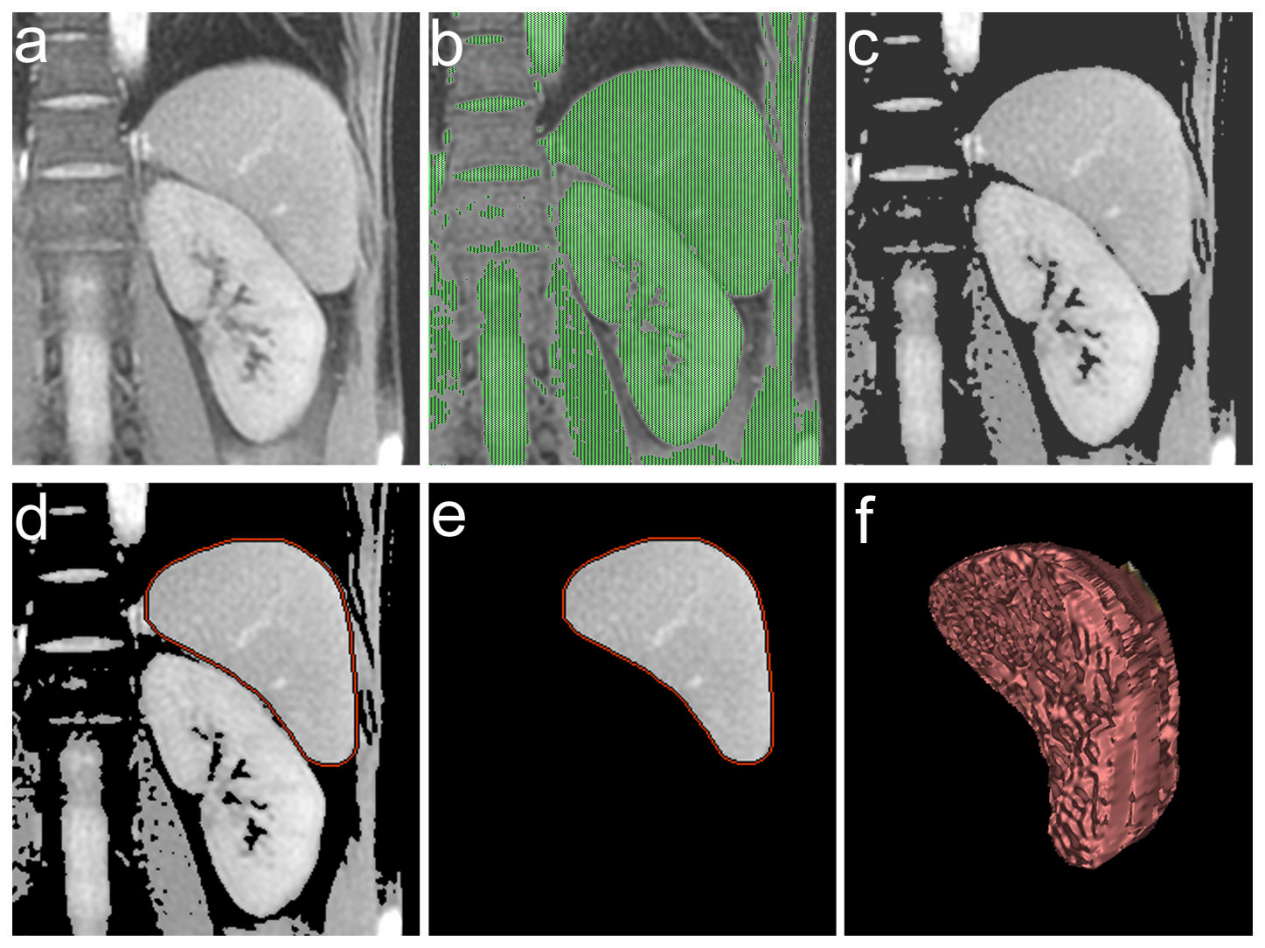
Spleen demarcation process. On each slice (a coronal image in this example) presenting the spleen (**a**), the operator defined a threshold signal intensity. Regions with signal intensities equal to or greater than the threshold were shaded green on the monitor of the workstation (**b**), and then selected (**c**). The operator manually demarcated the spleen on the images after thresholding, creating a red contour (**d**), and the spleen region was determined (**e**). The spleen was demarcated on all slices presenting the spleen. A volume-rendered image of the spleen is presented (**f**).

Spleen volume was measured from each image set. An operator demarcated the spleen using the volume utility on a workstation (Advantage Workstation; GE Healthcare). For demarcation, a thresholding method combined with manual processing was used (Figure 2). A threshold signal intensity was defined visually for each image set to separate the spleen from surrounding fat tissue. The spleen region was then selected manually from regions with signal intensities equal to or greater than the threshold, excluding adjacent soft tissues such as the stomach, left kidney, and abdominal wall. The spleen was demarcated on all slices presenting the spleen, and spleen volume was determined based on the sum-of-areas method.

Two spleen volumes estimated from the initial two image sets in the entire examination, which were acquired during free breathing, were averaged to determine the baseline spleen volume. The relationship between the two volumes was assessed by linear regression analysis. Moreover, the difference between the two volumes, the second volume minus the first volume, was plotted against the average. The bias was defined as the mean of the differences, and the 95% confidence interval was defined as the range of ±1.96 SD of the mean.

The control spleen volume for each respiratory manipulation was determined as the mean of two spleen volumes estimated from the control image sets, and normalized spleen volume was calculated as a percentage of the estimated spleen volume to the corresponding control spleen volume. Pulse rate and oxygen saturation were normalized similarly. The serial changes of the normalized values were evaluated. Additionally, the percentage of volume reduction was calculated from the mean value of five normalized spleen volumes during one respiratory manipulation. Spleen volume during respiratory manipulation in the unit of mL was calculated as the mean value of the five spleen volumes during one respiratory manipulation, and was compared with the control volumes.

### Statistical Analysis

 Values are presented as mean ± SD. Comparison was made by one-way analysis of variance followed by Fisher’s least significant difference test. Linear regression analysis was performed by the least squares method. A p value less than 0.05 was deemed statistically significant.

## Results

**Figure 3:**
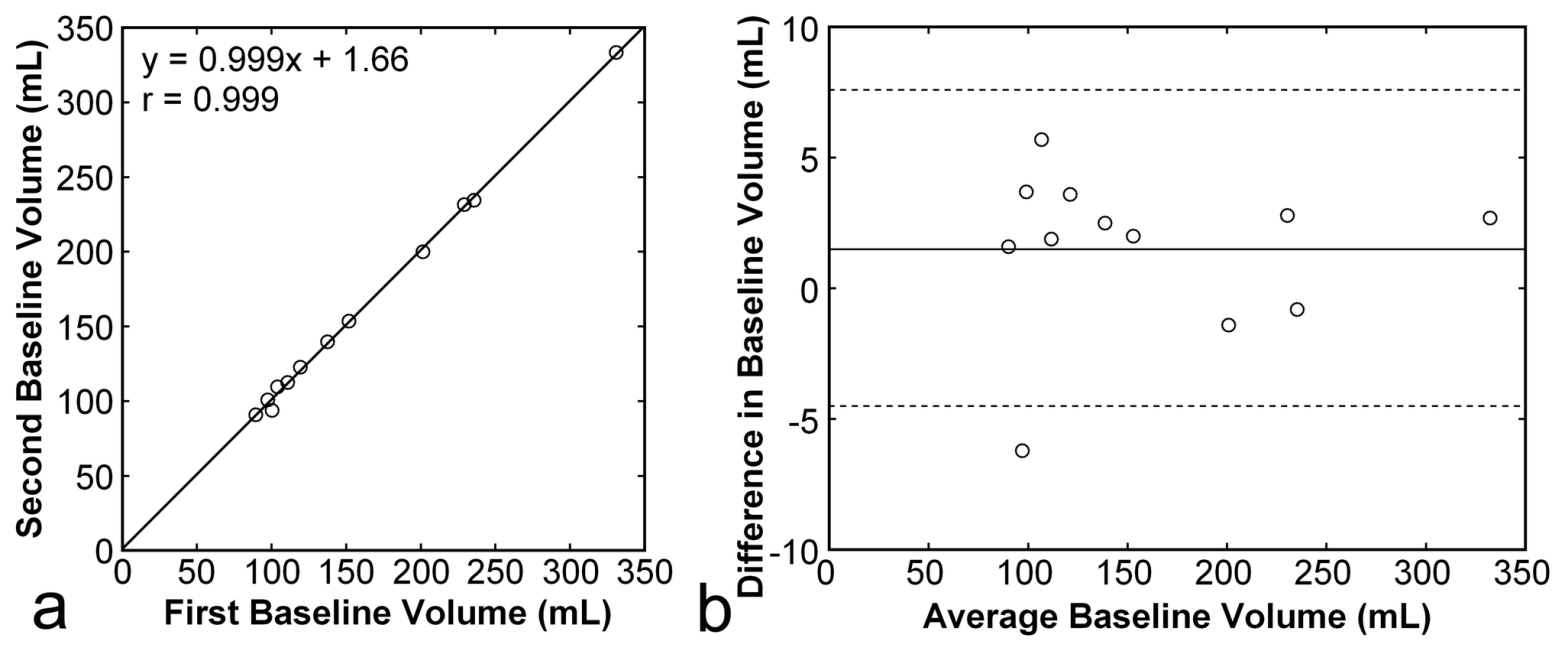
Repeatability of spleen volumetry. Baseline spleen volume measured from the second image set was plotted against that measured from the first image set (**a**). The solid line represents the regression line. The difference between the two baseline spleen volumes, the second volume minus the first volume, was plotted against the average of the two volumes (**b**). The solid line represents the mean of the differences (bias), and the broken lines represent the mean ± 1.96SD (95% confidence interval).

 All subjects successfully performed 30-s respiratory manipulations, including expiration breath holding, deep-inspiration breath holding, and the Valsalva maneuver. All image sets included the entire spleen and permitted volumetry of the spleen. Baseline spleen volume before respiratory manipulation was 173.0 ± 79.7 (range, 90.1–332.2) mL. The coefficient of variance for the two baseline volumes was 1.4% ± 1.6%. The two baseline volumes were closely correlated with a correlation coefficient of 0.999, and the difference between them was limited with a bias of 1.5 mL and a 95% confidence interval of -4.5 mL to 7.6 mL, suggesting excellent repeatability (Figure 3).

**Figure 4:**
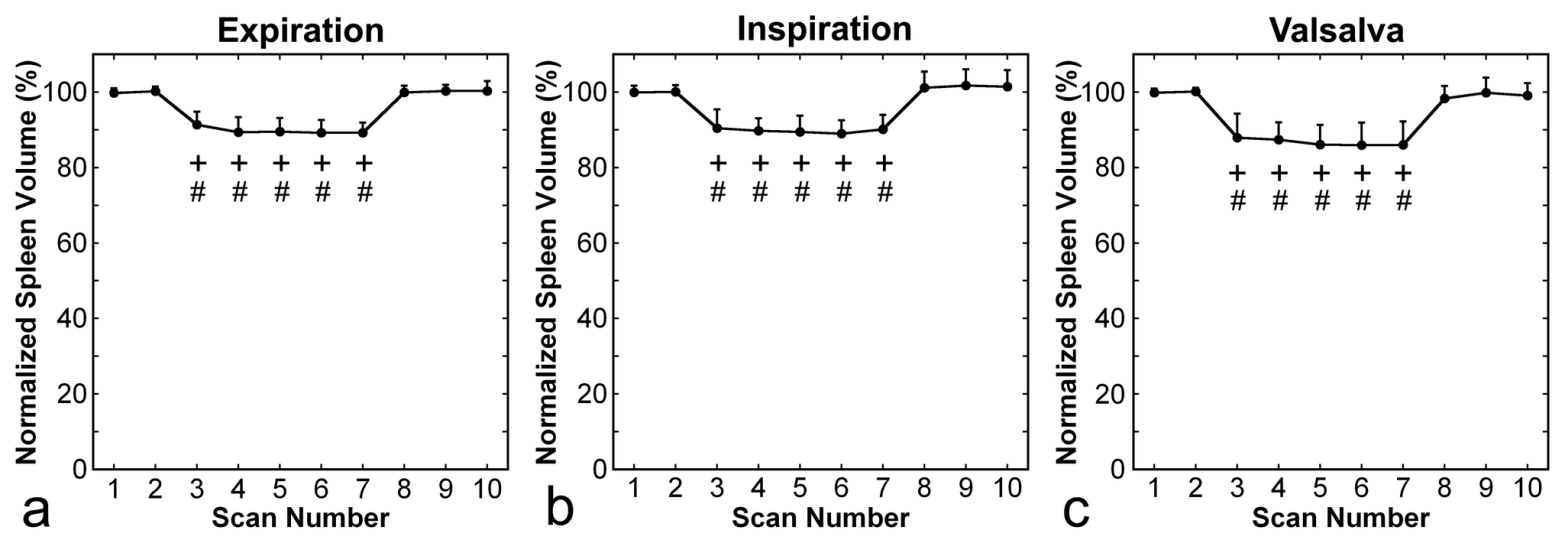
Time courses of normalized spleen volume. Panels **a**, **b**, and **c** present results for expiration breath holding, deep-inspiration breath holding, and the Valsalva maneuver, respectively. As shown in Figure 1, scans 3–7 were performed during respiratory manipulation, and the other scans were performed during free breathing. Error bars represent standard deviations. The + and # signs indicate statistically significant differences from control values obtained in scans 1 and 2, respectively.

**Figure 5:**
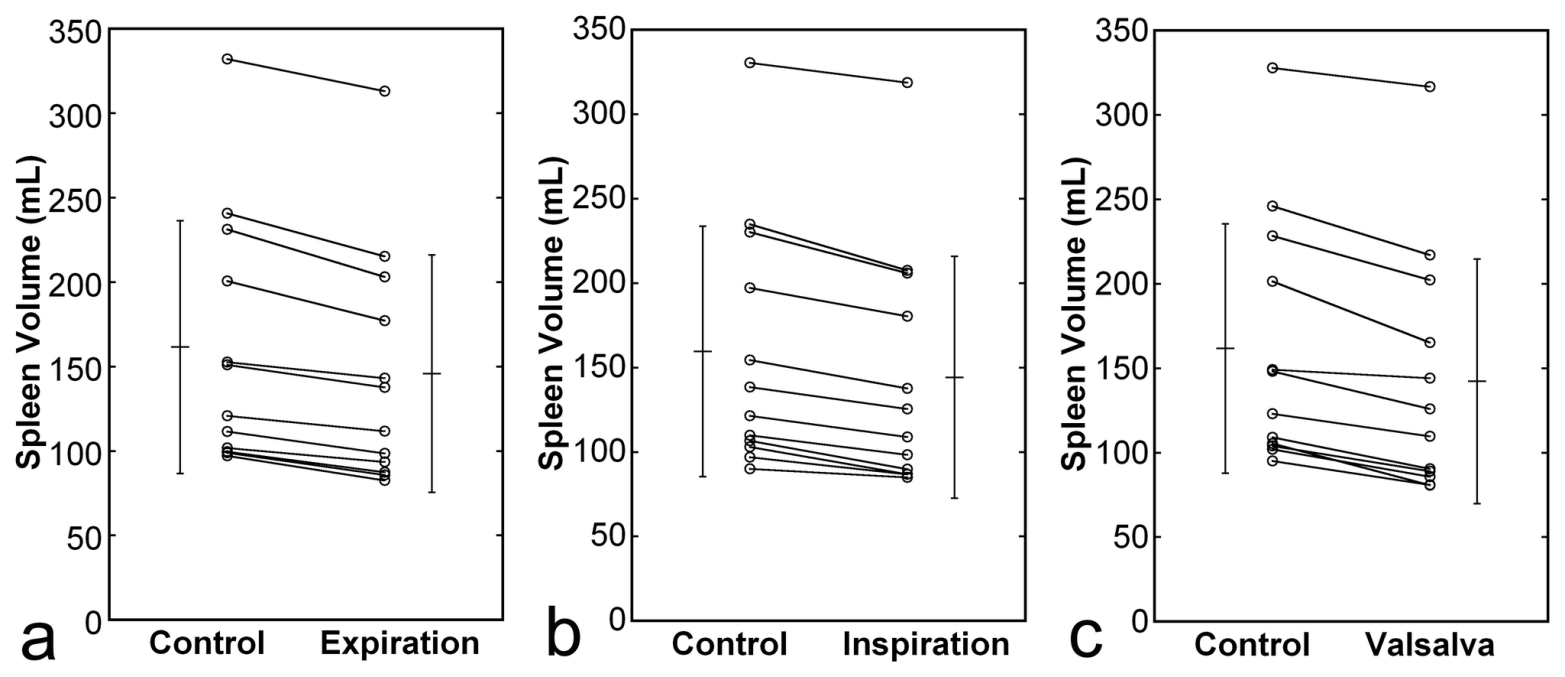
Changes in spleen volume during the respiratory manipulation. Panels **a**, **b**, and **c** present results for expiration breath holding, deep-inspiration breath holding, and the Valsalva maneuver, respectively. Plots indicate data for individual subject. Means and SDs are also presented.

 Reduction in spleen volume was observed during the respiratory manipulation, and the degree and time course of the volume change were similar irrespective of manipulation type (Figure 4). Normalized spleen volume decreased significantly just after commencement of the respiratory manipulation, remained constant during the manipulation, and returned to the control value 2 min after cessation of the manipulation. The percentages of volume reduction were 10.2% ± 2.9% (range, 5.7%–14.9%), 10.2% ± 3.5%(range, 3.5%–15.8%), and 13.3% ± 5.7%(range, 3.4%–23.5%) during expiration breath holding, deep-inspiration breath holding, and the Valsalva maneuver, respectively, and these values did not differ significantly. Volume reduction was shown for all subjects and all types of respiratory manipulations (Figure 5).

**Figure 6:**
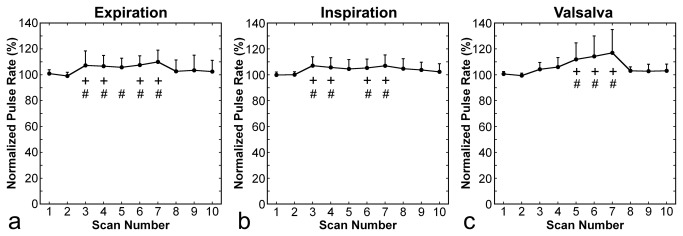
Time courses of normalized pulse rate. Panels **a**, **b**, and **c** present results for expiration breath holding, deep-inspiration breath holding, and the Valsalva maneuver, respectively. Error bars represent standard deviations. The + and # signs indicate statistically significant differences from control values obtained in scans 1 and 2, respectively.

**Figure 7:**
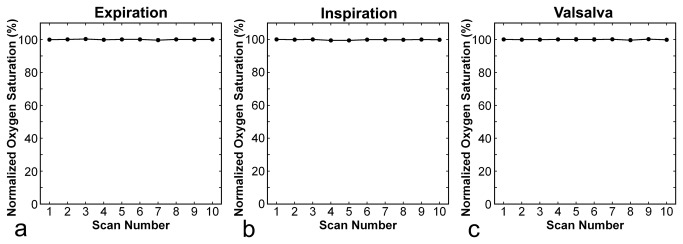
Time courses of normalized oxygen saturation. Panels **a**, **b**, and **c** present results for expiration breath holding, deep-inspiration breath holding, and the Valsalva maneuver, respectively. Error bars are not identifiable because of small standard deviations. No statistically significant difference from control values was found.

 The normalized pulse rate increased slightly during all types of respiratory manipulation, and returned to the control value 2 min after cessation of the manipulation (Figure 6). No decrease in normalized oxygen saturation was observed in relation to respiratory manipulations (Figure 7).

## Discussion

Ultrasonographic studies have demonstrated reductions in spleen volume in relation to breath-hold apnea [18–22]. The volume reduction has been speculatively attributed to spleen contraction *via* sympathetic activation [19,22–24], and mechanical compression may be also related [22]. Previous studies of spleen response to breath-hold apnea aimed to investigate physiology in diving, and the experimental conditions simulated diving. The spleen volume was reduced after repeated, long apnea dives in trained breath-hold divers, but not in untrained divers [18,21], although other studies have shown volume reduction in untrained subjects after repeated maximal apnea with [19] or without [20] face immersion in cold water. The spleen volume was measured after the end of breath-hold apnea in these studies but not during apnea [18–21], suggesting the persistence of volume reduction. Two studies specified the time course of recovery. The spleen volume returned to the level before breath holding 9 min after the end of the last apnea in one study [20] and did not completely recover, even at 60 min, in the other study [19]. Palada et al. [22] serially measured spleen volume before, during, and after short apnea in trained divers using ultrasonography through a fixed acoustic window. They found a 20% reduction during deep-inspiration breath holding and a 10% reduction during tidal-volume breath holding. Reduction was observed 3 s after the start of breath holding and lasted for 5 s after the end of breath holding. In addition, a study using radiolabeled erythrocyte and planar scintigraphic imaging showed a decrease in splenic erythrocyte content after 30-s breath holding, followed by recovery 2 min later, irrespective of diving experience [23]. The results of these studies employing short apnea [22,23] would be better applicable to usual measurement of spleen volume than those of other studies employing long apnea [18–21]. However, the reliability of ultrasonographic measurement of spleen volume without respiratory control appears to be unclear. Planar scintigraphic imaging is severely affected by attenuation of gamma rays in tissues and may cause errors due to changes in the spleen position.

In the present study, we repeatedly measured spleen volume in non-divers using MR imaging and demonstrated approximately 10% volume reduction during short breath holding, indicating a small but substantial influence of respiratory conditions on the estimated spleen volume. Spleen volumes measured during free breathing and breath holding are not exchangeable, and estimates should be presented with specification of the respiratory condition. In previous studies, the percentage of reduction ranged from 14% to 25% when reduction was observed [18–22]. The percentage of reduction was relatively small in the present study, which may have been due to less response in non-divers than in trained breath-hold divers. We used short breath-hold apnea, and the difference in severity of breath holding may also be responsible for this discrepancy.

Volume reduction was observed immediately after the start of respiratory manipulation and remained constant throughout the 30-s manipulation. This rapid response was consistent with the results of Palada et al. [22], who examined trained divers. The presence or absence of breath holding affects estimated spleen volume; however, the effect of the duration of breath holding appears to be negligible. Ultrasonographic measurement of spleen volume commonly requires a short breath hold, and most previous ultrasonographic studies of spleen volume may have been influenced by breath holding.

 In clinical settings, spleen volume may be measured from images obtained under various respiratory conditions. CT is commonly performed during deep-inspiration breath holding, and MR imaging is performed during expiration breath holding, deep-inspiration breath holding, or free breathing. Some patients may elevate intrathoracic pressure during deep-inspiration breath holding, similar to the Valsalva maneuver. The Valsalva maneuver increases intrathoracic and intra-abdominal pressure, reduces venous return, and stimulates sympathetic activity [25]. An 18% reduction in spleen volume has been demonstrated using ultrasonography during a 20-s Valsalva maneuver, without comparison with ordinary breath holding [24]. In the present study, the degree of reduction in spleen volume did not differ significantly among expiration breath holding, deep-inspiration breath holding, and free breathing, indicating that the type of respiratory manipulation does not substantially affect the estimated spleen volume. Palada et al. [22] demonstrated a larger reduction during deep-inspiration breath holding than during tidal-volume breath holding in trained divers, and commented that the difference may have been due to mechanical compression during deep-inspiration breath holding. Additional reduction related to mechanical compression may be present in trained divers but not in untrained subjects, which remains to be studied.

We allowed a 2-min interval between the end of respiratory manipulation and the first assessment of recovery to ensure regular respiration cycles during data acquisition, and revealed the resolution of volume reduction in the first assessment, in agreement with the findings of Palada et al. [22] after short apnea. Some studies [18,21] have observed no volume reduction in untrained subjects, which may have occurred because measurement was performed not during but after apnea in these studies. In clinical MR imaging of the abdomen, data acquisition is usually repeated during breath holding and free breathing. The respiratory condition during preceding imaging appears not to affect spleen volumetry during free breathing, at least, after an interval of 2 min. Because of the rapid, full recovery of spleen volume, we used spleen volumes measured 4 and 6 min after the end of respiratory manipulation as the control values for the evaluation of the subsequent respiratory manipulation session. Prolonged reduction in spleen volume was shown after repeated long apnea using ultrasonography [19,20], and the time course after intensive breath holding remains to be investigated using MR imaging.

 MR imaging provides morphological and functional measures with high accuracy and reproducibility under various conditions. Lack of radiation exposure facilitates repeated measurement and measurement in normal volunteers. MR imaging is a powerful tool for the investigation of *in vivo* physiology as well as for clinical diagnosis. MR imaging enables to measure spleen volume during free breathing with the aid of respiratory triggering and fast imaging techniques, and appears to be suitable for the assessment of spleen response to physiological stress. One slice was acquired for 266 ms at the end of expiration in one respiratory cycle in the present study, and the effect of movement during acquisition should be negligible. Stress-induced reduction in spleen volume has been speculated to be mediated by sympathetic stimulation [19,22–24] and may diminish in patients with impaired sympathetic nerve function. The spleen contains a large amount of blood cells and releases them on contraction. The ratio of the blood cell volume to the total splenic volume may vary in pathological conditions, presumably affecting contractile capacity. In addition, spleen stiffness increases in liver cirrhosis [26], which may disturb contraction. Responsiveness of the spleen in pathological conditions is a subject of future investigation.

## Conclusions

 The present MR study demonstrated that spleen volume is reduced during short breath-hold apnea. The presence or absence of breath holding is a major determinant of spleen volume, and the influences of the type of breath holding are limited. The response to breath holding is a rapid process. Spleen volume decreases just after the start of breath holding, and recovers soon after the end of breath holding. Physiological responses of the spleen to respiratory manipulations should be considered in the measurement and interpretation of spleen volume.
